# Examination of Multi-Receiver GPS/EGNOS Positioning with Kalman Filtering and Validation Based on CORS Stations

**DOI:** 10.3390/s20092732

**Published:** 2020-05-11

**Authors:** Adam Ciećko, Mieczysław Bakuła, Grzegorz Grunwald, Janusz Ćwiklak

**Affiliations:** 1 Faculty of Geoengineering, University of Warmia and Mazury in Olsztyn, 10-720 Olsztyn, Poland; mieczyslaw.bakula@uwm.edu.pl (M.B.); grzegorz.grunwald@uwm.edu.pl (G.G.); 2 Institute of Navigation, Military University of Aviation, 08-521 Deblin, Poland; j.cwiklak@law.mil.pl

**Keywords:** GPS, EGNOS, Kalman filter, multi-receiver

## Abstract

This paper presents the concept of precise navigation based on SBAS technology and CORS stations. In a kinematic test, three rover Global Positioning System (GPS) receivers, properly spaced relatively to each other, were used in order to estimate reliable and redundant GPS/EGNOS positions. Next, the Kalman filter was employed to give the final solution. It was proven that EGNOS positioning allows to obtain an accuracy in the range of about 0.5–1.5 m. The proposed solution involving the use of three mobile receivers and Kalman filtering allowed to reduce the 3D error to a level below 0.3 m. Such an accuracy was achieved using only GPS L1 code observations and EGNOS corrections. Additionally, a reliable monitoring of quality of GPS/EGNOS positioning in the test area based on CORS stations was presented.

## 1. Introduction

The accuracy and confidence level of the autonomous Global Positioning System (GPS) positioning are not satisfying for demanding safety of life (SoL) applications. Therefore, Satellite Based Augmentation Systems (SBAS) are used to improve the accuracy and integrity of GNSS (Global Navigation Satellite Systems) positioning. SBAS broadcasts corrections related to satellite positions and clocks as well as atmospheric delays (mainly ionospheric). The corrections data are transmitted to users via geostationary satellites. Therefore, SBAS receivers do not require extra hardware to use the correction signal [1]. EGNOS is one of the SBAS systems which supports the operation of GNSS [2]. Similar systems are being developed in the USA (WAAS—Wide Area Augmentation System), Japan (MTSAT—Satellite Based Augmentation System-MSAS), India (GAGAN—GEO Augmented Navigation System) and Russia (SDCM—System of Differential Correction and Monitoring). Currently the Russian SDCM is in the process of implementation. The European Space Agency (ESA), the European Commission (EC) and the European Organisation for the Safety of Air Navigation (EUROCONTROL) are involved in the EGNOS project [3]. The ESA is responsible for the design and development of the EGNOS system. International cooperation and coordination are the tasks of the European Commission. The EUROCONTROL defines the needs of civil aviation. The EGNOS works by transmitting the corrections and information about the failures of GPS satellites (in the future, also Galileo and GLONASS) via geostationary satellites. This makes it possible to increase the accuracy and integrity of navigation data, which is especially important in aviation [4,5,6,7] but can also be used in mobile mapping and remote sensing, maritime, rail and road, archaeology, geosciences and more [8,9,10,11,12,13]. EGNOS can be used in air transport in the case of approaches and landing procedures based on area navigation (RNAV) [14]. The non-precision approach (NPA) uses EGNOS for lateral guidance only. The approach with vertical guidance (APV-1) is an instrument procedure which does not meet the precision approach requirements but utilises lateral and vertical guidance. The basic premise of the EGNOS validation is to prove that this system can be used in SoL applications. This is possible if the EGNOS - SIS (Signal-In-Space) meets international requirements contained in the ESSC (EGNOS System Safety Case). EGNOS Open Service (which improves positioning accuracy) has been available since October 1, 2009. On the 2 of March 2011, the SoL service was officially launched. Its main objective is to support civil aviation operations according to Localiser Performance with Vertical Guidance (LPV) minima [15]. Current EGNOS performance is presented in the latest release of The European GNSS Agency document from 2019 [15]. Positioning accuracy in this document is determined by the 3 m (95%) in the horizontal plane and 4 m (95%) in a vertical plane. It should be stressed that these values are established for the whole area covered by the operation of EGNOS. Until recently, the lack of ranging and integrity monitoring station (RIMS) stations to the east of Warsaw (central Poland) resulted in a poor accuracy and integrity of positioning in Poland as well as in the eastern part of Europe [16]. However, the latest research on the EGNOS system in Ukraine shows that despite the absence of an RIMS station on that territory, the integrity and effectiveness of EGNOS positioning in this country has been recently greatly improved [11].

Modifications of standard solutions based on SBAS systems have already been the subject of many studies. The results of research on improving the integrity and availability of SBAS positioning are reported in [17,18,19]. There were also studies in which the use of non-standard EGNOS ionospheric models were examined and evaluated [20,21].

SBAS systems can also be an alternative to the post-processing technique for the purpose of GNSS augmentation [22]. The latest practical tests of GPS/EGNOS positioning in central Europe showed that the horizontal accuracy of a single EGNOS receiver is in the range of 0.5–2.5 m and the vertical one: 1.5–3.0 m [23,24]. The motivation of this paper is to present a method of increasing EGNOS accuracy by using three rover GPS receivers. These receivers are placed at fixed distances between each other [25,26,27]. Relatively fixed distances between rover receivers improve not only the accuracy but also the reliability of GPS kinematic positioning, which is indispensable in applications devoted to air navigation. An additional independent method of checking the integrity of the GNSS solution in a test area is presented in Section 5. Additional validation of the quality of GNSS positioning is based on information from the Continuously Operating Reference Stations (CORS). This test can be utilised to detect local gross errors related to, e.g., multipath, jamming and spoofing.

## 2. GPS/EGNOS Measurements and Errors Characteristics 

Normally, all GPS techniques use code or/and carrier phase measurement. Both can be used to measure distances (pseudorange) between GNSS receivers located on Earth and satellites. For each measurement to a satellite, code range may be modelled [28] as follows:

(1) $$P\left(t\right)=\rho \left(t\right)+cdt\left(t\right)-cdT\left(t\right)+d_{ION}\left(t\right)+d_{TROP}\left(t\right)+d_{EPHEM}\left(t\right)+d_{P}\left(t\right)$$

where P(t) is the measurement pseudorange in meters, ρ(t) is the true receiver-to-satellite geometric range in meters, c is the speed of light in meters per second, dt(t) is the satellite clock error in seconds, dT(t) is the receiver clock error in seconds, $d_{ION}\left(t\right)$ is the ionospheric delay error in meters, $d_{TROP}\left(t\right)$ is the tropospheric delay error in meters, $d_{EPHEM}\left(t\right)$ is the delay error in meters due to the satellite ephemeris error, and $d_{P}\left(t\right)$ represents other pseudorange errors in meters, such as multipath, interchannel receiver biases, thermal noise, receiver and satellite hardware delay, as well as pseudorange measurement noise. The pseudorange would equal the geometric distance if the propagation medium was a vacuum and if there were no clock errors or other biases [29].

Code measurements are commonly used in GPS/EGNOS positioning. The general observation model of coordinates in GPS/EGNOS positioning may be expressed as follows:

(2) $$y=Y+\delta \left(l_{1},l_{2},\ldots l_{n_{l}}\right)+\delta_{0}+g+\varepsilon$$

where the quantity $\delta \left(l_{1},l_{2},\ldots l_{n_{l}}\right)$ constitutes a deterministic error, dependent on the finite number of variables $l_{1},l_{2},\ldots l_{n_{l}}$. These variables in GNSS positioning are caused by the delays of ionosphere and troposphere, ephemeris data errors, satellite clock errors and GPS receiver clock errors. Through $\delta_{0}$, we denote a systematic error which is constant for its value and sign. In this context, the deterministic error is frequently named a variable systematic error. The g and ε quantities are random measurement errors [30]. Although the errors are similar in character, their origin and interpretation are quite different. The g error is a random error, which is difficult to predict and also difficult to accept (often called gross error). The ε error is also a random error but its value is rather steady and the value of this error is specific to a particular GNSS positioning method. Every GNSS measurement method carries random errors (ε) which are impossible to predict. 

However, the application of the advanced determinist error models does not free the observations from random errors in the GNSS navigation. This is because in GNSS navigation, using EGNOS, the positions to be determined are based on code measurements, which have large random errors. Therefore, using additional GNSS receivers on a mobile platform may significantly decrease the random errors as well as improving the integrity of EGNOS positioning, particularly in air navigation. The issue of supporting GNSS positioning with SBAS systems has been the subject of many previous studies [31,32,33]. In the approach proposed in this article, the computations are made in a “position domain” and SBAS positioning is combined both with Kalman filtering and the use of a multi-receiver solution.

## 3. Kalman Filtering of EGNOS Positions in Multi-Receiver Positioning Results

**Figure 1:**
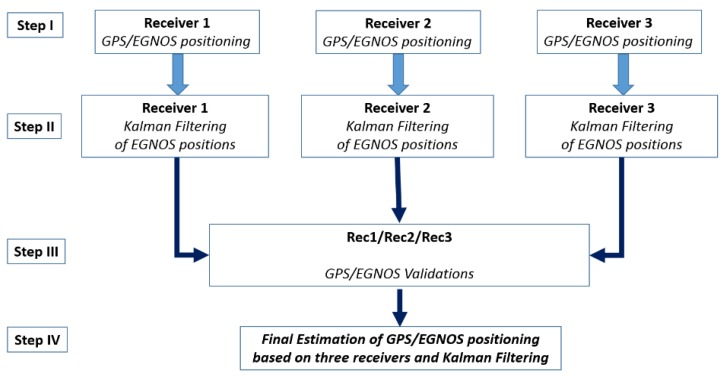
Flow chart of EGNOS positioning by means of three GPS/EGNOS receivers.

Let us assume that the coordinates in the global International Terrestrial Reference Frame (ITRF) (φ—*ellipsoidal latitude*, λ—*ellipsoidal longitude* and h—*ellipsoidal height*) are subjected to measurement, using the EGNOS correction. Therefore, for the determined coordinates, it is possible to assign an elementary functional model:

(3) $$\begin{array}{c} \hat{\varphi }\left(t\right)=\varphi \left(t\right)+\Delta \varphi \left(t\right)\\ \hat{\lambda }\left(t\right)=\lambda \left(t\right)+\Delta \lambda \left(t\right)\\ \hat{h}\left(t\right)=h\left(t\right)+\Delta h\left(t\right) \end{array}$$

where Δφ, Δλ, Δh are the values of overall EGNOS positioning errors for the particular horizontal components (Δφ, Δλ) and the vertical one (Δh). Due to the fact that the GPS-EGNOS positions carry a certain random error, the values of Δφ, Δλ, Δh may vary slightly in successive measurement epochs and it is difficult to estimate them precisely. Therefore, for each GPS/EGNOS receiver, we used the Kalman filter in order to dampen random errors influencing the determined coordinates. Kalman filtering is a popular method used as an enhancement in various GNSS applications [34,35]. Determining the position of a mobile platform is realised in several stages (Figure 1):

**I.** Independent positioning of GPS/EGNOS rover receivers, determination of geodetic coordinates of every receiver (*Rec1*, *Rec2*, *Rec3*), in the world geodetic frame —ITRF:

$\varphi_{Rec1},\varphi_{Rec2},\varphi_{Rec3}$—ellipsoidal latitude in ITRF frame
$\lambda_{Rec1},\lambda_{Rec2},\lambda_{Rec3}$—ellipsoidal longitude in ITRF frame
$h_{Rec1},h_{Rec2},h_{Rec3}$—ellipsoidal height in ITRF frame

For the simplicity of further calculation, in this stage of the process, the φ and λ coordinates were converted to rectangular system of coordinates [36]. In the following stages of calculation, the *N*, *E* and *h* coordinates are used.

**II.** Filtering of EGNOS coordinates for each receiver independently, based upon EGNOS positions of each receiver and the Kalman filter. For all receivers, the input for position determination was: GPS L1 raw observation data with 1 s interval, augmented with EGNOS data. EGNOS messages include accurate information on the position of each GPS satellite, the accuracy of the atomic clocks on board the satellites and up-to-date information about the ionosphere. The filtering was made according to the following scheme [28]:

- Computation of Kalman gain:
  (4) $$K_{k}=P_{k}^{-}H_{k}^{T}{\left(H_{k}P_{k}^{-}H_{k}^{T}+R_{k}\right)}^{-1}$$
- Update of estimate with new measurement:
  (5) $$x_{k}=x_{k}^{-1}+K_{k}\left(z_{k}-H_{k}x_{k}^{-1}\right)$$
- Computation of error covariance for updated estimate:
  (6) $$P_{k}=\left(I-K_{k}H_{k}\right)P_{k}^{-1}$$
- Calculation of values in advance:
  (7) $$x_{k+1}^{-}=\Phi_{k}x_{k}$$
  (8) $$P_{k+1}^{-}=\Phi_{k}P_{k}\Phi_{k}^{T}+Q_{k}$$
  where
  $K_{k}$—Kalman gain;
  $P_{k}$—predicted value of estimation covariance;
  $H_{k}$—observation matrix;
  $P_{k}^{-}$—corrected value of estimation covariance;
  $Q_{k}$—covariance of dynamic disturbance noise;
  $R_{k}$—covariance of measurement uncertainty;
  $\Phi_{k}$—state transition matrix;
  $x_{k}^{-1}$—predicted value of the estimated state vector;
  $x_{k}$—corrected value of the estimated state vector;
  $z_{k}$—observation vector of EGNOS positioning.

The efficiency of Kalman filtering is mostly dependent on the mathematical observation model, which determines the relationship between observations and parameters [28], i.e.,

(9) $$z_{k}=H_{k}x_{k}+v_{k}$$

and the adopted dynamic model, i.e.,

(10) $$x_{k+1}=\Phi_{k}x_{k}+w_{k},$$

(11) $$E\left[v_{k},v_{i}^{T}\right]=\{\begin{array}{c} R_{k},\;i=k\\ 0,\;i\neq k \end{array}$$

(12) $$E\left[w_{k},w_{i}^{T}\right]=\{\begin{array}{c} Q_{k},\;i=k\\ 0,\;i\neq k \end{array}$$

where

$z_{k}$—observation vector;
$v_{k}$—the noise of the observation model;
$w_{k}$—the noise of the kinematic model.

In this research, Kalman filtering was applied to horizontal and vertical coordinates, which were determined by EGNOS positioning. The horizontal and vertical coordinates were further processed independently of each other both for rover receivers, placed on the car, and for GPS receivers, located at reference stations. Therefore, an identical kinematic and observational model was used, both for mobile and static receivers. This is possible by using pseudo-observation in EGNOS positioning for each of the components Δn(t),Δe(t),Δh(t), which are estimated using formula (13).

(13) $$\begin{array}{c} \Delta n\left(t\right)=N\left(t\right)-N{\left(t\right)}_{EGNOS}\\ \Delta e\left(t\right)=E\left(t\right)-E{\left(t\right)}_{EGNOS}\\ \Delta h\left(t\right)=h\left(t\right)-h{\left(t\right)}_{EGNOS} \end{array}$$

where

N(t), E(t), h(t)—reference coordinates with centimetre accuracy (about 1–2 cm) derived from RTK postprocessing technique;
$N{\left(t\right)}_{EGNOS}$, $E{\left(t\right)}_{EGNOS}$, $h{\left(t\right)}_{EGNOS}$—EGNOS positions in subsequent measurement epochs.

The value $v_{k}$, understood as the noise of the observation model, is assumed at the level of 1.75 m, which is related to the accuracy of the code positioning for the L1(GPS) frequency. The idea of the Kalman’s filtration is, among other things, the right choice of the variance of the kinematic model—$w_{k}$ (also understood as the noise of the model), which determines the accuracy of the model. For the purpose of this research, for the speed of 5 km/h and measurement, every 1 s, the assumed $w_{k}$ value was set at 0.1 m. However, if the speed was increased to, i.e., 200 km/h, this value would have to be much higher. Therefore, for different speeds and types of movement, the $w_{k}$ value must be adapted based on previous experimental data sets.

Based on the adopted pseudo-observation model, the $H_{k},$ $\Phi_{k}$, $R_{k}$,$\;Q_{k}$ matrices are therefore equal, respectively:

(14) $$H_{k}=\left[\begin{array}{ccc} 1 & 0 & 0\\ 0 & 1 & 0\\ 0 & 0 & 1 \end{array}\right]$$

(15) $$Q_{k}=\left[\begin{array}{ccc} 1 & 0 & 0\\ 0 & 1 & 0\\ 0 & 0 & 1 \end{array}\right]$$

(16) $$R_{k}=\left[\begin{array}{ccc} 1.75^{2} & 0 & 0\\ 0 & 1.75^{2} & 0\\ 0 & 0 & 1.75^{2} \end{array}\right]$$

(17) $$Q_{k}=\left[\begin{array}{ccc} 0.1^{2} & 0 & 0\\ 0 & 0.1^{2} & 0\\ 0 & 0 & 0.1^{2} \end{array}\right]$$

while the observation vector will contain psudo-observations (13), i.e.,

(18) $$z_{k}=\left[\begin{array}{c} \Delta n\\ \Delta e\\ \Delta h \end{array}\right].$$

The relationships between the $z_{k}$ and $x_{k}$ vectors are specified in formulas (9) and (10), and are dependent on a’priori acquired noise of the observation model ($v_{k}$) and noise of the kinematic model ($w_{k})$.

**Figure 2:**
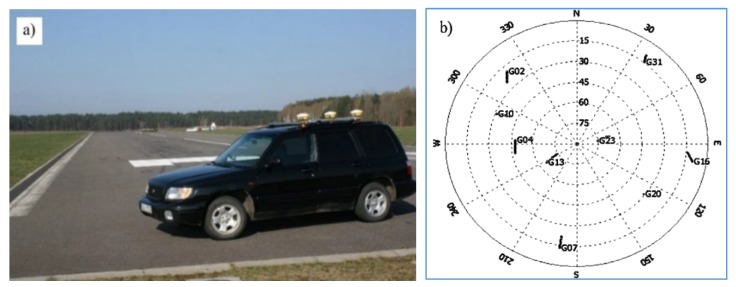
Test area: (**a**) airfield runway with a mobile GNSS unit; (**b**) satellite configuration during the experiment.

**III.** Validation of the determined EGNOS positioning computed in stage II. At this stage of the process, the consistency of the results obtained from three independent receivers was determined. It was achieved by means of the known geometrical conditions due to the placement of all three receivers. The receivers were put in a single line on the roof of the car (see Figure 2a) with known fixed distances, which means that the left side of the formula (19) theoretically is equal to 0. The consistency of the results was determined according to the following formula:

(19) $$\left[\begin{array}{c} \Delta n_{V}\left(t\right)\\ \Delta e_{V}\left(t\right)\\ \Delta h_{V}\left(t\right) \end{array}\right]=2^{-1}\left[\begin{array}{c} N_{Rec1}\left(t\right)+N_{Rec3}\left(t\right)\\ E_{Rec1}\left(t\right)+E_{Rec3}\left(t\right)\\ h_{Rec1}\left(t\right)+h_{Rec3}\left(t\right) \end{array}\right]-\left[\begin{array}{c} N_{Rec2}\left(t\right)\\ E_{Rec2}\left(t\right)\\ h_{Rec2}\left(t\right) \end{array}\right]$$

where

Δ*n_V_*, Δ*e_V_*, Δ*h_V_*—difference of *N*, *E*, *h* coordinates, calculated by means of the geometrical placement of three receivers;
$N_{Rec1},\;N_{Rec2},\;N_{Rec3}$—northing coordinate of receiver 1, 2 and 3;
$E_{Rec1},\;E_{Rec2},\;E_{Rec3}$—easting coordinate of receiver 1, 2 and 3;
$h_{Rec1},\;h_{Rec2},\;h_{Rec3}$—height of receiver 1, 2 and 3.

**IV.** Estimation of the position of a vehicle ($N_{Rover},\;E_{Rover},\;h_{Rover}$) which is the centre-point of a geometrical figure, composed of three GPS/EGNOS receivers. In the first step of this stage, position was estimated as an average value of the positions from three independent receivers calculated in previous stages. It was computed according to the following formula:

(20) $$\left[\begin{array}{c} N_{Rover}\left(t\right)\\ E_{Rover}\left(t\right)\\ h_{Rover}\left(t\right) \end{array}\right]=3^{-1}\left[\begin{array}{c} N_{Rec1}\left(t\right)+N_{Rec2}\left(t\right)+N_{Rec3\;}\left(t\right)\\ E_{Rec1}\left(t\right)+E_{Rec2}\left(t\right)+E_{Rec3}\left(t\right)\\ h_{Rec1}\left(t\right)+h_{Rec2}\left(t\right)+h_{Rec3}\left(t\right) \end{array}\right]$$

In the second step of this stage, the final position of a vehicle was determined with the use of Kalman filter according to formulas (4)–(8). The input for this filtering were coordinates obtained from Equation (20). Although the final solution is burdened with all GNSS positioning errors, it should be emphasized that the impact of errors through the applied algorithm was reduced. The reduction was due to averaging of three independent solution and double Kalman filtering.

## 4. Performance of Multi-Receiver EGNOS Positioning in a Test Area 

Kinematic test measurements were conducted at a civilian airport (Figure 2a) in Olsztyn (North-Eastern Poland). The test involved three GPS receivers, which were placed on the roof of a car. They were mounted in the line along the movement of the car. The main receiver (*Rec.2*) was placed in the middle, and the two auxiliary receivers (*Rec.1, Rec.3*) were located on both sides of the main receiver at a constant distance of 0.5 m. The length of the mobile platform is about 1 m, so it is possible to mount it in the cabin of the airplane for flight experiments in the future. In the test measurements, the car with three GPS receivers made two full laps along the external line of a runway. During the measurement, there were seven GPS satellites available above 15 degrees elevation of (Figure 2b). They were used to calculate GPS position, taking into account the EGNOS corrections. 

The kinematic GNSS measurements were taken with Trimble SPS 882 receivers. The SPS 882 is a flexible site positioning system available to heavy highway and marine construction contractors. The SPS 882 delivers unmatched power, accuracy and performance in a rugged, compact unit that can stand up to the harsh conditions typically found on construction sites. Raw GPS observations were registered for three rover receivers and a reference station with one second interval. For the sake of estimation of EGNOS positioning, only GPS code observations at L1 frequency were used along with EGNOS corrections provided by ESA. The computations were done with RTKLIB software—the open source program package for GNSS positioning (www.rtklib.com). The further calculations were executed using self-developed script in MATLAB. Moreover, the kinematic measurements were also made with the use of GPS phase measurements by means of a reference station located at a distance of approximately 2 km from the runway. This allowed for the calculation of the accurate reference positions of the mobile receivers. The exact coordinates obtained from the phase positioning, with centimetre-level accuracy, calculated according to formula (20), served as a reference in further analyses of the EGNOS positioning. The errors of the reference coordinates were calculated according to

(21) $$\left[\begin{array}{c} {\Delta n}_{V,RTK}\left(t\right)\\ {\Delta e}_{V,RTK}\left(t\right)\\ {\Delta h}_{V,RTK}\left(t\right) \end{array}\right]=2^{-1}\left[\begin{array}{c} N_{Rec1,RTK}\left(t\right)+N_{Rec3,RTK}\left(t\right)\\ E_{Rec1,RTK}\left(t\right)+E_{Rec3,RTK}\left(t\right)\\ h_{Rec1,RTK}\left(t\right)+h_{Rec3,RTK}\left(t\right) \end{array}\right]-\left[\begin{array}{c} N_{Rec2,RTK}\left(t\right)\\ E_{Rec2,RTK}\left(t\right)\\ h_{Rec2,RTK}\left(t\right) \end{array}\right]$$

**Figure 3:**
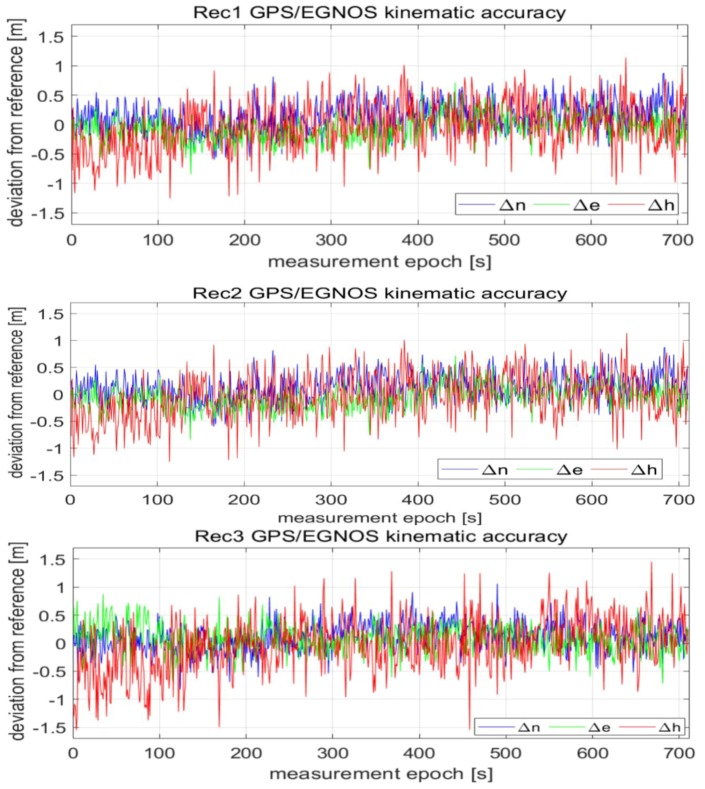
Accuracies in GPS kinematic positioning of individual receivers, by means of EGNOS correction.

The errors of reference values of $\Delta n_{V,RTK},\Delta e_{V,RTK},\Delta h_{V,RTK}$ computed in the Universal Transverse Mercator (UTM) system ranged from −12 mm up to 9 mm for the horizontal coordinates (*North (N)*, *East (E)*), and from −16 mm up to 22 mm for the vertical coordinate *(H)*. Determination of the exact coordinates (2–3 cm of accuracy) of the mobile devices (*Rec1, Rec2, Rec3*) allowed for the next step of analyses. In the following phase of work, the GPS/EGNOS positioning was analysed in detail. To this end, EGNOS corrections, provided by the service called EGNOS Message Server (EMS), were used. The EMS service continuously stores the SBAS messages broadcast by the EGNOS system in hourly text files [37]. For each mobile GPS unit, the computation of horizontal and vertical positions was carried out in UTM system. The results of the deviation of the horizontal (Δn,Δe) and vertical (Δh) positions for the individual receivers were calculated as the differences between reference coordinates and GPS/EGNOS coordinates, as depicted in Figure 3.

**Figure 4:**
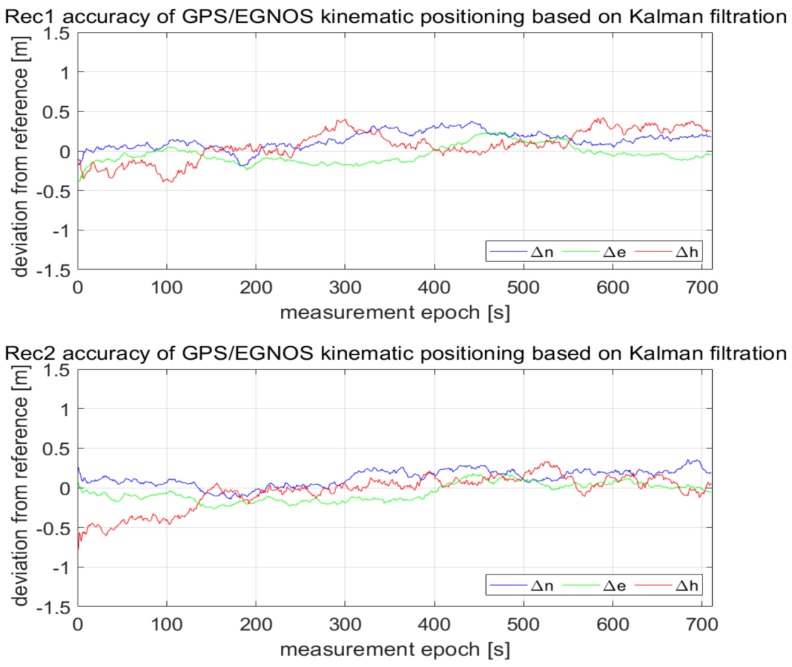

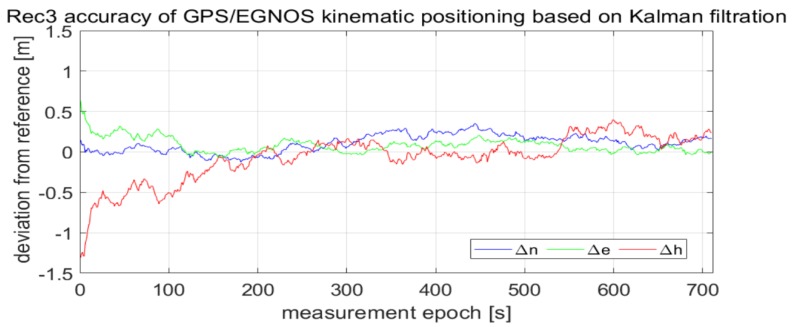
Accuracies of GPS kinematic positioning of individual receivers, by means of EGNOS correction and Kalman filtering.

**Figure 5:**
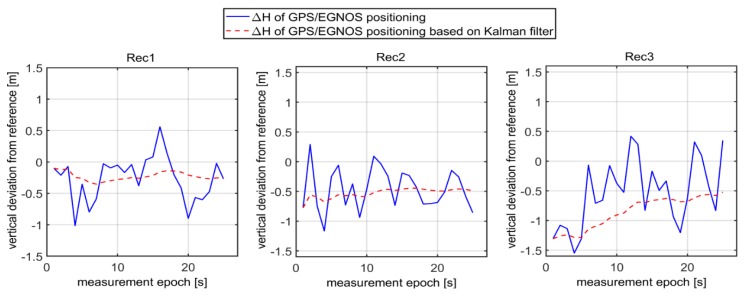
Accuracy of the vertical coordinate of EGNOS positioning for three mobile receivers in initial measurement time span together with Kalman filtering.

As can be seen in Figure 3, the values of Δn,Δe,Δh representing deviations of horizontal and vertical positions of GPS/EGNOS positioning in relation to precise phase positioning fluctuated in the range of around ± 1.5m. In the next stage, Kalman filtering of the coordinates obtained from the individual GPS/EGNOS receivers was accomplished. The results are presented in Figure 4, which clearly illustrates the considerable dampening of random errors due to the use of the Kalman filter. It is also visible that receiver No. 3 (Figure 4) had significantly larger deviations of the vertical coordinate at the beginning of its operation. This led to noticeable deterioration of the position compared to the other two GPS receivers, since with the Kalman filter, the initial positions already had big errors. The next analysis, presented graphically in Figure 5, shows that the altitude deviation of the EGNOS position for receiver No. 3 was quite large in the beginning of the observation. The EGNOS position errors in Figure 5 were obtained by subtracting RTK results from EGNOS results. The error value was at the level of 1.0–1.5 m for the first 6 s, which directly affected Kalman filter results. 

Presumably, the value of altitude error of the receiver No. 3 in the initial measurement time span (Δh) was caused by some systematic factors, since its character is not random (Figure 5). Therefore, the values of the altitude of receiver No. 3 were burdened with the systematic error, which would be impossible to detect by a single GPS receiver. Therefore, using two additional GPS receivers (*Rec.1* and *Rec.2*), whose altitude errors in the initial time span are depicted in Figure 5, caused considerable suppression of altitude errors in the final result. According to Figure 1, the third step of the process is validation of EGNOS positioning based on three GPS receivers, which is conducted according to formula (21). 

**Figure 6:**
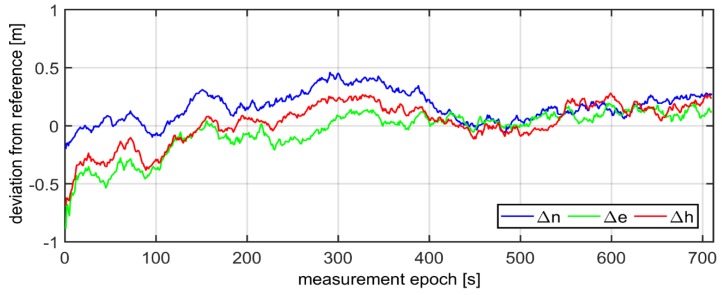
Estimated EGNOS positioning errors determined through internal validation of kinematic positioning, for horizontal coordinates (Δn,Δe) and the vertical coordinate (Δh).

In such a situation, the values $\Delta n_{V},\;\Delta e_{V},\;\Delta h_{V}$ calculated on the basis of formula (21) would have an independent and reliable validation of SBAS positioning, in their character. In the test calculations, only GPS satellites were used, since at present, the EGNOS data contain corrections for the GPS system only. The results of the GPS/EGNOS system validation, based on three receivers, are depicted in Figure 6.

**Figure 7:**
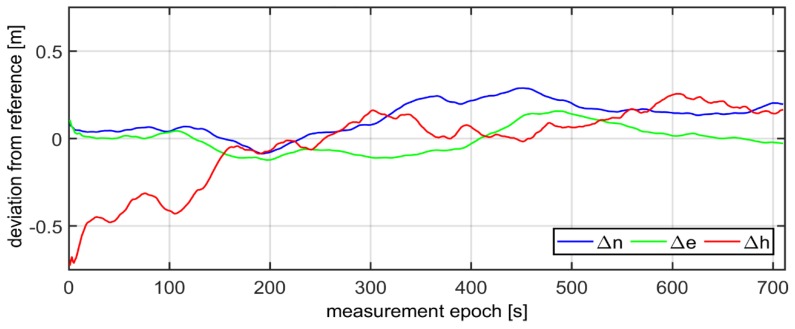
True errors of final GPS-EGNOS positioning by means of three GPS receivers and Kalman filter.

Having conducted the validation of the coordinates by means of three GPS receivers, the fourth step of the diagram presented in Figure 1, the coordinates, which refer to the geometrical centre-point of the three mobile receivers, were calculated on the basis of formula (20). Next, the Kalman filtering of the coordinates of centre-point was performed. The results of the final deviations of the coordinates in the kinematic positioning, using three GPS/EGNOS receivers, are shown in Figure 7. All computations were made in a “position domain” and therefore, no separate error components were taken into consideration. The total error was assumed as one value with the influence of all factors on accuracy of positioning. The input for this filtering were only the coordinates obtained from Equation (20). One can notice that in general, the errors of horizontal and vertical positioning (Δn, Δe,Δh) did not exceed 0.3 m. Moreover, it is possible to observe that for the vertical coordinate, initial errors were bigger due to much higher systematic errors. Nevertheless, after the subsequent time measurement span, the Kalman filter tremendously smoothed the error of the altitude coordinate. It must be stressed that the horizontal coordinates error was below 0.2 m from the very beginning of the test.

The vertical coordinate in GNSS positioning techniques is usually burdened with the biggest errors, which adversely affect air navigation. Although the final solution is affected with all GNSS positioning errors, it should be stressed that the impact of errors through the applied algorithm has been greatly reduced. The presented approach depicts that there are various possibilities of enhancing the accuracy of positioning, which exploit independent measurement methods.

## 5. GPS-EGNOS Integrity Monitoring Based on Continuously Operating Reference Stations (CORS)

In this part of the article, a concept of additional authentication of the results is introduced. The process presented in this section allows the user to perform additional validation of the final solution based on information from the CORS stations. In the air navigation, a GPS/EGNOS receiver located on board of a plane has no on-line connection with ground permanent GNSS stations. Currently, in many countries, there are networks of permanent GNSS stations at a distance of at most 70 km, which are used for ground DGNSS measurements. Although they are used primarily for ground RTK measurements and the collection of GNSS observations for various post-processing calculations, they may also be used to monitor the accuracy of the SBAS system. Generally, large systematic errors in SBAS positioning are principally eliminated by Receiver Autonomous Integrity Monitoring (RAIM) algorithms, which use redundant observations in order to detect signal irregularities with special software [38]. This does not mean, however, that gross errors are completely eliminated, due to many external factors, such as multipath or intentional interference of GNSS signals. 

**Figure 8:**
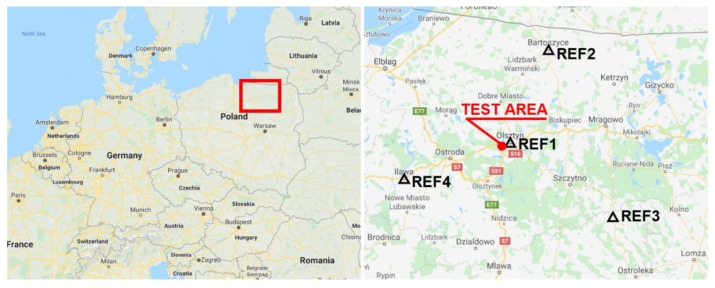
Location of the test area and CORS stations around the test area.

The actual positioning accuracy of the SBAS system in a given area can, however, be examined using CORS permanent reference stations. Calculating the GPS/EGNOS position in real time on a CORS station with known coordinates (e.g., station REF1), using formula (13), we can determine the actual horizontal (Δn,Δe) and vertical (Δh ) positioning errors of EGNOS in a given area. This is due to the fact that for fixed reference stations, the N(t), E(t), h(t) coordinates are invariable in subsequent measurement epochs and equal to the coordinates of the reference stations. In a practical static test, four equally spaced CORS stations, located around the airfield were used. One of these stations, REF1 was at a distance of 5 km form the test site, while the other three stations (REF2, REF3, REF4) were evenly distributed at a distance of about 60–80 km (Figure 8).

**Figure 9:**
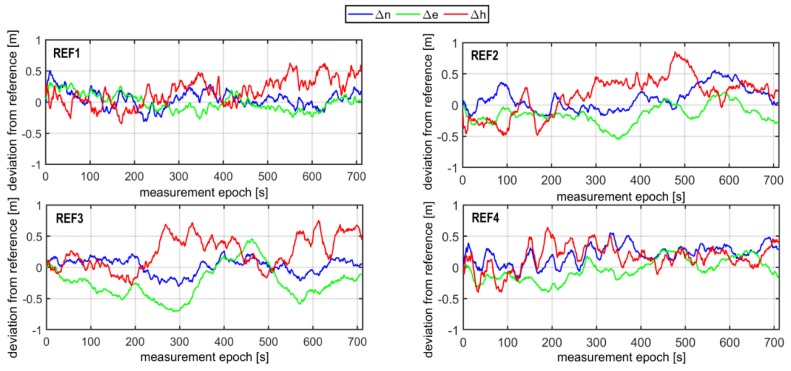
The GPS-EGNOS positioning accuracy at 4 permanent ASG-EUPOS stations, located around the airfield, based on GPS L1, EGNOS and Kalman filtering.

The REF1 station was equipped with Javad Delta-3 multi-GNSS reference station (GPS+GLONASS+Galileo+BeiDou). Javad Delta-3 is a powerful and reliable receiver for high-precision navigation systems; it can also operate as a CORS or a portable base station for RTK applications. The other stations were equipped with Trimble NetRS GPS-only receivers. The Trimble NetRS GPS receiver is designed for use as a CORS for geodetic, survey, high-accuracy GIS and monitoring applications. To determine the GPS/EGNOS position for each reference station, GPS L1 code observations and EGNOS corrections were used. In addition, Kalman filtering of the results from CORS stations was carried out in accordance with previously presented formulas (4)–(8). All computations were made in “position domain”. The positioning errors achieved for the time period corresponding with the kinematic measurements are shown in Figure 9. The values of these errors both for a horizontal and vertical position oscillated in a range of up to 0.8 m from the true values.

Although simultaneous analysing of the results of multiple CORS reference stations is quite troublesome, it is possible to determine the median of the real horizontal and vertical positioning errors. The median is the measure known as large systematic errors resistant and therefore, the more reference stations, the better. However, in addition to monitoring of these errors expressed by the median, additional information about the occurrence of gross error at any reference station is needed. The median does not depend on extreme values. This information can be very important in the context of a precise and reliable navigation. Therefore, a measure of the reliability of the selected CORS stations can be given in a simple statement:

(22) $$\Delta_{Me-X}\left(t\right)=Me\left(t\right)-\bar{x}$$

where Me(t)—the median of errors calculated on the basis of CORS stations and $\bar{x}$—arithmetic mean of these errors calculated on the basis of the same CORS station. 

**Figure 10:**
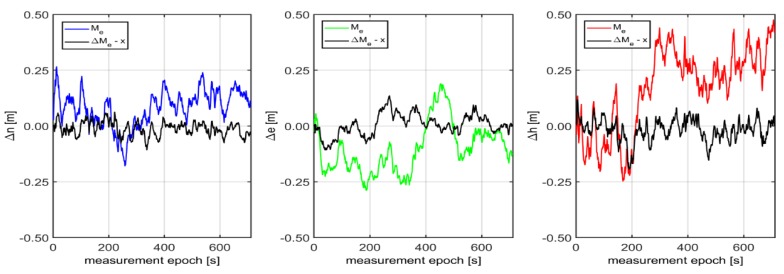
The median Me(t) values and the $\Delta_{Me-X}\left(t\right)$ values based on observations at the stations: REF1, REF2, REF3 and REF4 calculated with the use of the Kalman filter.

The above expression of the difference between the median and the mean value informs about large systematic errors. If the error occurs at any CORS station, the simple arithmetic average is not immune to gross errors and therefore, the $\bar{x}$ value will deviate from the Me(t). Therefore, if gross errors occur, the $\Delta_{Me-X}\left(t\right)$ value will significantly deviate from zero. However, if big systematic errors do not occur, the $\Delta_{Me-X}\left(t\right)$ value will be around zero. The process of revealing the gross errors in GNSS positioning results can be performed with the use of data collected from nearby CORS stations. This calculation can expose the local gross errors related to, e.g., multipath, jamming or spoofing. This information can be of great value for safety-of-life applications. The median values and the $\Delta_{Me-X}\left(t\right)$ values based on observations at the stations REF1, REF2, REF3 and REF4 are shown in Figure 10, where the calculated horizontal and vertical components of GPS/EGNOS positioning accuracy can be seen more clearly than those shown in Figure 9 for individual CORS stations. In addition, the oscillation of $\Delta_{Me-X}\left(t\right)$ in the range of zero informs about the lack of significant gross errors in determining the GPS/EGNOS position during the experiment. According to the authors’ opinion, such calculations could be made in a ground monitoring centre operating on the basis of the Ground Based Augmentation System (GBAS). If any irregularities were detected, such a system would inform the pilot of the possible impact of the error on the satellite navigation system.

## 6. Summary and Conclusions

This paper presents a concept of using the SBAS technology for augmented aerial navigation, which may be used for both aircraft and unmanned aerial vehicles. The conducted experiments confirm that three GPS receivers properly spaced relatively to each other and used for kinematic navigation can significantly reduce random errors. Using three independent GPS receivers largely increases the reliability and accuracy of SBAS positioning. In the test measurements, the initial accuracy of GPS/EGNOS positioning was in the range of 0.5–1.5 m. The proposed solution involving the use of three mobile receivers and Kalman filtering allowed to reduce the 3D error to a level below 0.3 m. This accuracy was achieved using only GPS L1 code observations and EGNOS corrections. 

Unfortunately, this solution does not provide important information about the global accuracy of the GPS/EGNOS positioning in the given area. This problem can be overcome with the use of existing networks of CORS GNSS stations. Although these networks are devoted mainly to surveying, they can provide vital information concerning the integrity of EGNOS positioning and can be used to monitor the accuracy of real-time GNSS positioning in the region. 

The EGNOS positioning model presented in the article is based on multiple GPS receivers and it can be used in aerial navigation. By combining this model with the Inertial Navigation System (INS), it can further improve the accuracy and integrity of precision navigation. In that situation, we can use information from the gyroscope and the accelerometer and use these additional observations in the Kalman filter algorithm. Therefore, the authors plan to conduct further research in order to perform hybrid navigation, both in post-processing and for real-time applications. 

The most relevant outcome of this research is the confirmed increase of accuracy and integrity of positioning based on multi-receiver GPS/EGNOS positioning with Kalman filtering and validation based on CORS stations. In the near future, the authors are planning to perform flight experiments, which could be an important step towards the implementation of this solution into air navigation.
